# Co-alterations associated with resistance to EGFR inhibitors in advanced EGFR-positive NSCLC: insights from the precision oncology program in two large international real-world cohorts

**DOI:** 10.1038/s41698-026-01617-5

**Published:** 2026-07-22

**Authors:** LA Boos, C. Doerig, E. Camarillo-Retamosa, M. Sarwary, S. Rizzo, G. Gut, N. Miglino, L. Fábregas-Ibáñez, C. Früchtenicht, M. Zoche, B. Bodenmiller, S. Chevrier, AS Eklund, M. Nowak, S. Rahmani-Khajouei, GC Berardo, L. Kaczmarek, K. Bosshard, W. Archey, M. Bodmer, D. Glinz, I. Opitz, S. Ulrich, M. Guckenberger, L. Hempel, I. Stiefel, P. Rahimzadeh, B. Gosztonyi, U. Richter, L. Bankel, A. Wicki

**Affiliations:** 1 Department of Medical Oncology and Hematology, University Hospital Zurich, Zurich, Switzerland; 2 Faculty of Medicine, University of Zurich, Zurich, Switzerland; 3 F. Hoffmann-La Roche AG, Basel, Switzerland; 4 Genentech Inc, South San Francisco, CA USA; 5 Pathology - Molecular Tumor Profiling, University Hospital, Zurich, Switzerland; 6 Department of Quantitative Biomedicine, University of Zurich, Zurich, Switzerland; 7 Institute of Molecular Systems Biology, Federal Institute of Technology Zurich, ETH, Zurich, Switzerland; 8 Navignostics AG, Horgen, Switzerland; 9 Department of Thoracic Surgery, University Hospital Zurich, Zurich, Switzerland; 10 Department of Pulmonology, University Hospital Zurich, Zurich, Switzerland; 11 Department of Radiooncology, University Hospital Zurich, Zurich, Switzerland

**Keywords:** Biomarkers, Cancer, Oncology

## Abstract

EGFR inhibitors (EGFRi), alone or combined with chemotherapy or bispecific antibodies, are standard first-line therapy for advanced EGFR-mutant NSCLC, but their efficacy is limited by heterogeneous resistance mechanisms. To define the impact of genetic co-alterations, we analyzed two complementary real-world cohorts. Patients with EGFR exon 19 deletion or L858R mutations were included from the University Hospital Zurich (USZ; *n* = 43) and the Flatiron Health–Foundation Medicine clinicogenomic database (FH-FMI CGDB; *n* = 1927). Duration of treatment (DOT) on first-line EGFRi was assessed in relation to co-alterations at diagnosis. In the FH-FMI CGDB, shorter DOT on osimertinib was associated with co-alterations in ERBB2 (*p* < 0.01), PIK3CA (*p* = 0.04), and TP53 (*p* < 0.01), with similar trends for MET, KRAS, BRAF, and CCNE1. For erlotinib/afatinib, shorter DOT was observed for ERBB2 (*p* = 0.027) and TP53 (*p* < 0.01), alongside additional genes. The USZ cohort showed concordant trends. TP53 was the most frequent co-alteration; variants affecting zinc-binding and dimerization domains were associated with shorter DOT. These findings identify co-alterations linked to reduced EGFRi benefit, particularly ERBB2, PIK3CA, and TP53, and highlight residue-specific TP53 effects. This supports comprehensive genomic profiling and function-based stratification to refine treatment strategies in EGFR-mutant NSCLC.

## Introduction

Activating alterations in the kinase domain of the epidermal growth factor receptor (*EGFR*) are a frequent oncogenic driver in non-small cell lung cancer (NSCLC). Up to 15% of NSCLC in Caucasians and up to 50% in East Asians harbor oncogenic *EGFR* alterations, with a high incidence in never-smokers or former light smokers and females^1,2^. Two EGFR alterations are frequently detected and referred to as the most common (“classic”) alterations in NSCLC: deletions in the *EGFR* exon 19 and the L858R point mutation in exon 21 (*EGFR*^+^)^2,3^. Over the last decade, EGFR tyrosine kinase inhibitors (EGFRi) have become the standard first-line treatment for advanced *EGFR*^+^ NSCLC, with osimertinib currently representing the preferred option due to its favorable efficacy and safety profile^4,5^. Over time, resistance against EGFRi occurs in the vast majority of patients. Mechanisms include mutations within the EGFR gene itself (on-target) and activation of alternative pathways (off-target)^6–8^.

Identifying patients at risk of EGFRi resistance is of utmost importance, as additional treatment options have recently been introduced for the first-line palliative treatment of advanced *EGFR*^+^ NSCLC. The combination of osimertinib with chemotherapy and the combination of amivantamab (a bispecific antibody targeting *EGFR* and the mesenchymal-epithelial transition (*MET*) receptor) with lazertinib (a potent third-generation EGFRi) have become standard of care first-line treatment options^9,10^. However, while the combination of treatments in the FLAURA 2 and the MARIPOSA trials can achieve higher efficacy, they can also be associated with higher toxicity and a need for more and longer health care visits, which may therefore impact patients‘ quality of life. Therefore, the identification of patients benefitting from combination treatment or osimertinib treatment alone has become increasingly important. Certain high-risk features were identified in the study populations of these large regimen-introducing studies, such as detectable baseline ctDNA, *TP53* co-mutation and liver and brain metastases, which could be stratification factors for patient selection^9,10^. However, further insights are needed to integrate this risk stratification into daily cancer care.

*TP53* is the most frequently mutated gene in human cancer, with alterations occurring in more than half of all primary tumors^11,12^. The zinc-binding domain, due to its central role in maintaining p53 functionality, has become the focus of intense research aimed at elucidating its pathogenic relevance and exploring therapeutic strategies to restore zinc-dependent structural integrity^13,14^.

Mutations in *TP53* also represent the most frequent co-alteration in *EGFR*^+^ NSCLC in particular^12,15^. Multiple studies have demonstrated that *TP53* mutations are associated with inferior clinical outcomes and reduced sensitivity to EGFRi^16,17^. In particular, mutations located within the exons 5–8 encoding the DNA-binding domain of *TP53*, were associated with worse prognosis^18^. Specific mutations, such as *R248Q*, *R273H*, and *R175H*, have been implicated in promoting resistance to the third-generation EGFRi Osimertinib^17^. Importantly, a retrospective real-world study conducted in an Asian cohort suggested that intensified treatment strategies may improve outcomes in patients with *EGFR*^+^ NSCLC and *TP53* co-alterations^19^. Despite these insights, data on the prognostic impact of specific *TP53* mutations—particularly at the exon or functional domain or single amino acid level—remain limited. This lack of granularity restricts the clinical utility of *TP53* status as a robust biomarker for treatment escalation or de-escalation strategies in *EGFR*^+^ NSCLC.

Furthermore, clinical trial populations are often highly selected by eligibility criteria, thus, not fully representing patients in the real-world setting. This is particularly relevant for advanced *EGFR*^+^ NSCLC, as a recent real-world study showed that 55% of patients treated with first-line osimertinib would not have met the eligibility criteria for the FLAURA trial^20^. This underscores the growing complexity of first-line treatment decision-making for advanced *EGFR*^+^ NSCLC and highlights the need for additional stratification parameters to more accurately classify patients into higher- and lower-risk subpopulations, thereby supporting more individualized therapeutic choices. Early analyses from the MARIPOSA trial reported lower frequencies of on-target resistance mechanisms and MET amplifications for patients treated with the combination of amivantamab and lazertinib compared to those receiving Osimertinib^21^. This showcases how treatment choices influence resistance mechanisms and emphasizes the need to consider individual patient characteristics when selecting first-line treatment.

First and second-generation EGFRi are used in uncommon *EGFR* alterations, for compound alterations in the *EGFR* gene and later-line treatment in advanced *EGFR*^+^ NSCLC^4^. However, data for these approaches remain limited, and the role of early-generation EGFRi will be redefined in a setting with increasing treatment possibilities.

The Precision Oncology Program (POP), a public-private partnership project (University Hospital of Zurich (USZ), University of Zurich (UZH) and Hoffmann-La Roche AG) has provided insights into the potential of data- and profiling-driven decision support for medical oncology, hypothesizing that real-world data (RWD) and Imaging Mass Cytometry (IMC) can further inform individualized treatment recommendations in cancer care^22^. The presented study includes two complementary RWD sources: the USZ POP database as a small, clinically well-characterized cohort providing descriptive case-level context, and the Flatiron Health-Foundation Medicine Clinicogenomic Database (FH-FMICGDB) as a large clinicogenomic dataset enabling broader explorative analyses^23^. Within this framework, we assessed the association between *EGFR*-relevant co-alterations involved in *EGFR*-associated signaling pathways and treatment outcomes in advanced *EGFR*^+^ NSCLC across different EGFRi treatments. A particular focus was placed on TP53 alterations, which were analyzed at the functional domain and residue level to address the limited granularity of existing evidence^16,17,19^.

## Results

### Patient cohorts, clinical characteristics, and treatment patterns

Analyses in the FH-FMI CGDB cohort were performed to assess associations between co-alterations and treatment duration, while findings in the USZ cohort are reported descriptively.

**Fig. 1:**
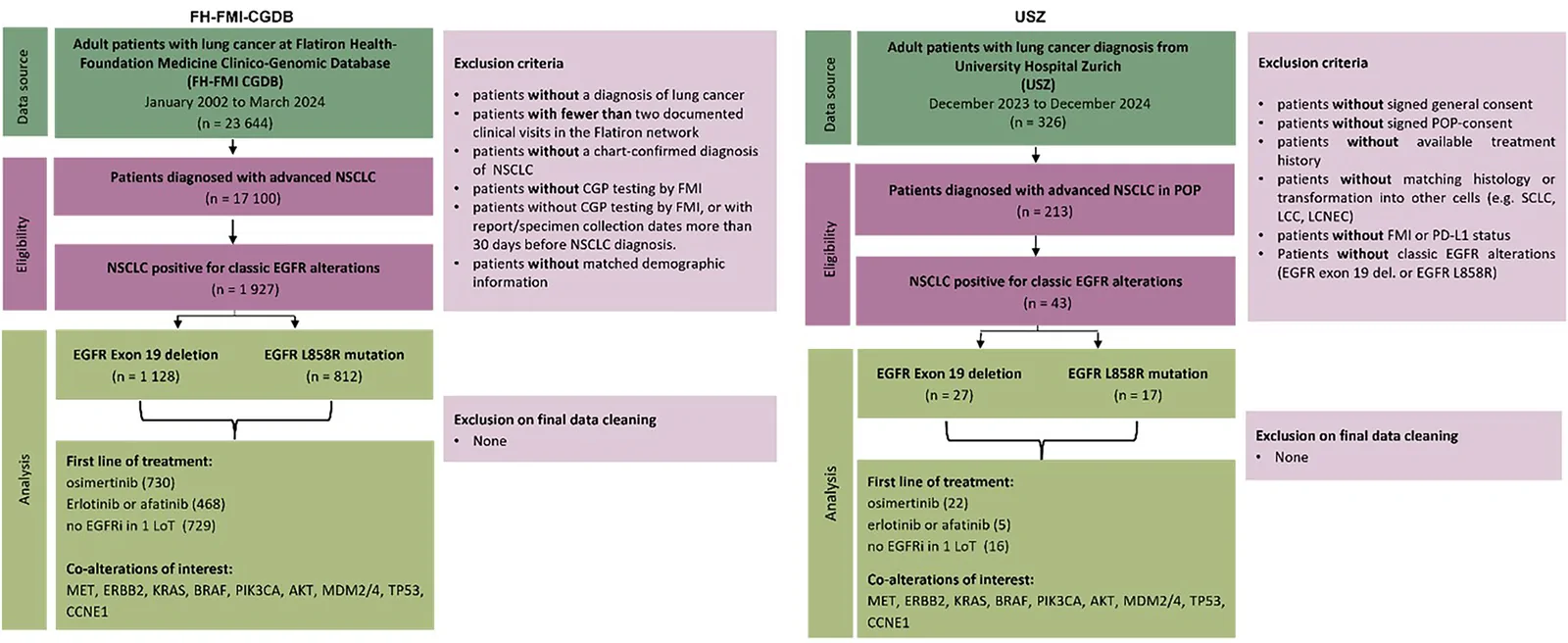
Patient selection and case inclusion process. The ESMO-Oncology Real-World Evidence (GROW) chart provides an overview of the data selection and case inclusion process, illustrating the flow of patient assortment according to the key decision points, co-alterations and EGFRi treatment. The patients were stratified based on the data source (dark gray boxes), eligibility criteria (dark pink boxes) for the analysis (light green box) of classic EGFR alteration, EGFR exon 19 deletion and EGFR L858R mutation with first-line treatment either with osimertinib or erlotinib/afatinib and the presence or absence of genetic co-alterations. The flowchart also includes the analysis’s exclusion criteria (light pink boxes).

A total of 43 patients with advanced *EGFR*^+^ NSCLC were enrolled in the POP study at USZ, and 1,927 patients with advanced *EGFR*^+^ NSCLC were selected from the FH-FMI CGDB cohort based on predefined matching criteria (Fig. 1). The demographic characteristics of both cohorts are presented in Table 1. Notable differences were observed, including a higher median age and a greater proportion of patients with ECOG ≥ 2 in the FH-FMI CGDB cohort. The FH-FMI CGDB cohort contained more patients with locally advanced disease (16.3% vs. 2.3%). PD-L1 status was more often unknown in the FH-FMI CGDB cohort (PD-L1 unknown 44% vs. 0%). The FH-FMI CGDB cohort included a limited number of squamous cell carcinoma (37 cases) (Table 1). First-line treatment differed between the two cohorts, reflecting the different timeframes of SACT administration and evolving treatment practices. USZ patients – treated between October 2014 and May 2025 – received predominantly EGFRi. Patients within the FH-FMI CGDB database - treated between January 2002 and March 2024 with treatment initiation predominantly in more recent years–received a broader distribution of first-line treatments, including EGFRi and chemotherapy-based regimens, with osimertinib representing the most frequently used individual EGFRi in both cohorts. (Supplementary Table 1; Supplementary Fig 1).

**Table 1:** Demographic, clinical, and molecular characteristics of the two study cohorts

	FH-FMI-CGDB (*n* = 1927)	USZ (*n* = 43)
Age Median (range)	73 (65–81)	67 (57–75)
Sex at birth		
F	1270 (66%)	25 (58%)
M	657 (34%)	18 (42%)
Smoking status		
History of smoking	838 (43%)	22 (51%)
No history of smoking	1086 (56%)	19 (44%)
Unknown/Not documented	*	2 (5%)
Cancer Stage		
Stage III	16 (0.8%)	-
Stage IIIA	195 (10%)	1 (2.3%)
Stage IIIB	106 (5.5%)	-
Stage IIIC	*	-
Stage IV	1242 (64%)	19 (44.2%)
Stage IVA	182 (9.4%)	9 (21%)
Stage IVB	181 (9.4%)	14 (32.5%)
Histology		
Non-squamous cell carcinoma	1866 (97%)	43 (100%)
NSCLC histology NOS	24 (1.2%)	-
Squamous cell carcinoma	37 (1.9%)	-
Molecular testing		
Tissue based	1241 (64.4%)	34 (79%)
Liquid biopsy based	182 (9.4%)	3 (7%)
Tissue and liquid biopsy based	504 (26.2%)	6 (14%)
PD-L1		
PD-L1 > 50%	201 (10%)	5 (12%)
PD-L1 1-49%	347 (18%)	16 (37%)
PD-L1 < 1%	383 (20%)	22 (51%)
PD-L1 unknown or unvaluable	996 (52%)	-
ECOG		
0–1	1141 (59%)	42 (98%)
≥2	195 (10%)	1 (2%)
Unknown	591 (31%)	-
Site of metastasis	***N*** = **3156**	***N*** = **105**
Brain	558 (17%)	13 (12%)
Liver	238 (8%)	7 (7%)
Other	2360 (75%)	85 (81%)

The table presents the characteristics of USZ and FH-FM-CGDB patients. Cases under each category are designated in number (n) and the frequency (%). Site of metastasis “other” included the localization at the adrenal gland, bone, lymph nodes, kidney, lung, pancreas, peritoneum, pleura, skin, soft tissue, spleen, thyroid. Asterisk (*) designates cohorts with six or fewer patients omitted from the FH-FMI CGDB cohort, dash (-) no reported cases.

### Association of TP53 co-alterations with DOT in *EGFR*^+^ NSCLC on EGFRi in the first treatment line

**Fig. 2:**
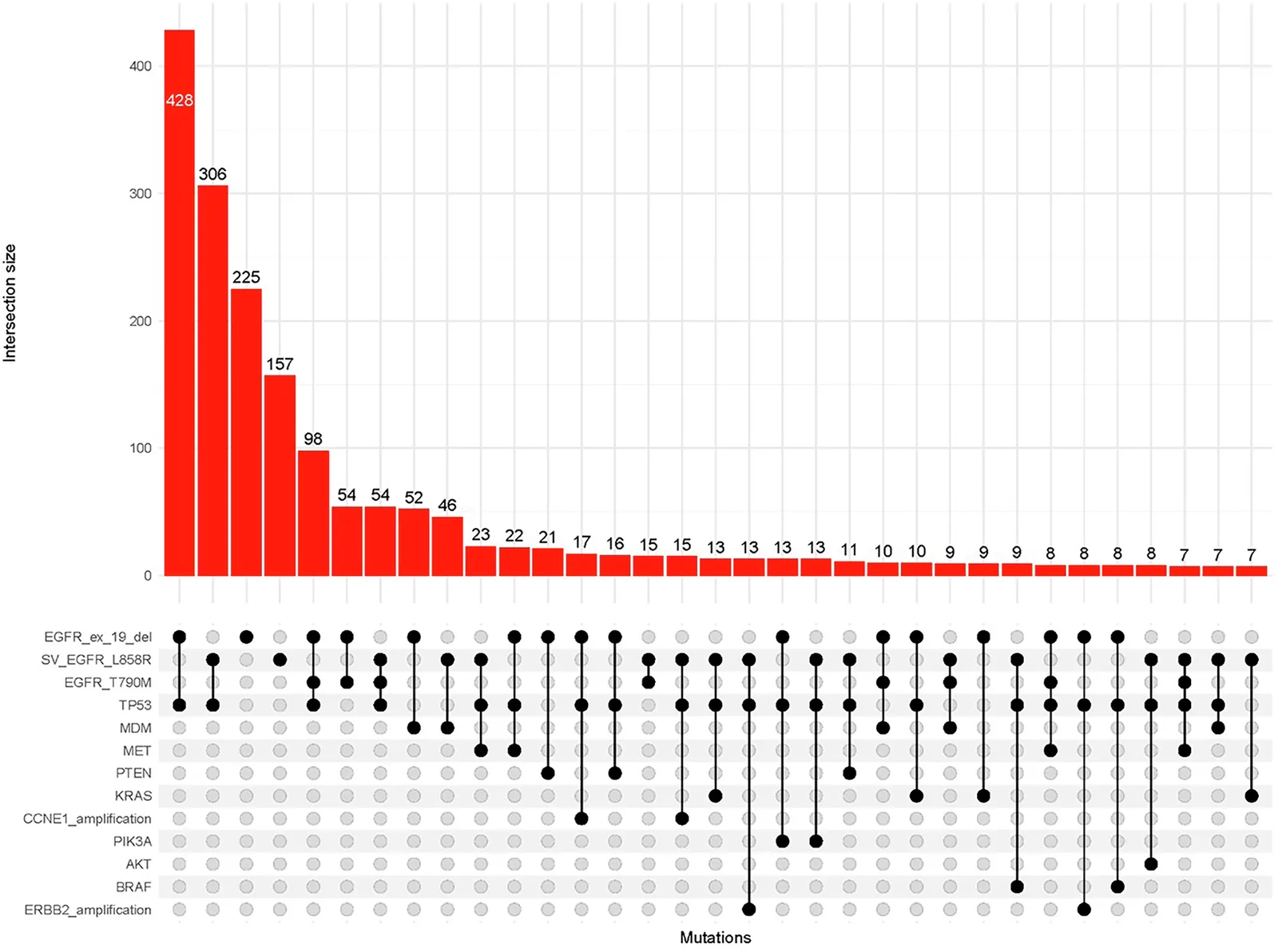
Co-occurrence patterns of genomic alterations in advanced EGFR^+^ NSCLC in the merged study cohort. The UpSet plot illustrates the patterns of co-alterations among cancer-related genes of interest in the merged study cohort combining FH-FMI CGDB and USZ patients. Red vertical bars show the number of cases for each specific combination of mutations, and the dark connected dots below the bar graph indicate the genes involved in co-occurrence.

In 18/43 (42%) of USZ patients with advanced *EGFR*^+^ NSCLC, one or more co-alterations of interest were identified in the standard-of-care molecular pathological testing. 1590/1927 (82.5%) patients in the FH-FMI CGDB cohort had one or more co-alterations of interest. The most common co-alteration across both cohorts was *TP53* (17/18 cases (94.4%) USZ cohort; 1091/1590 cases (68.7%) FH-FMI CGDB cohort), which co-occurred more frequently with an *EGFR* exon 19 deletion than with an *EGFR L858R* mutation (Fig. 2 and Supplementary Fig. 2).

**Fig. 3:**
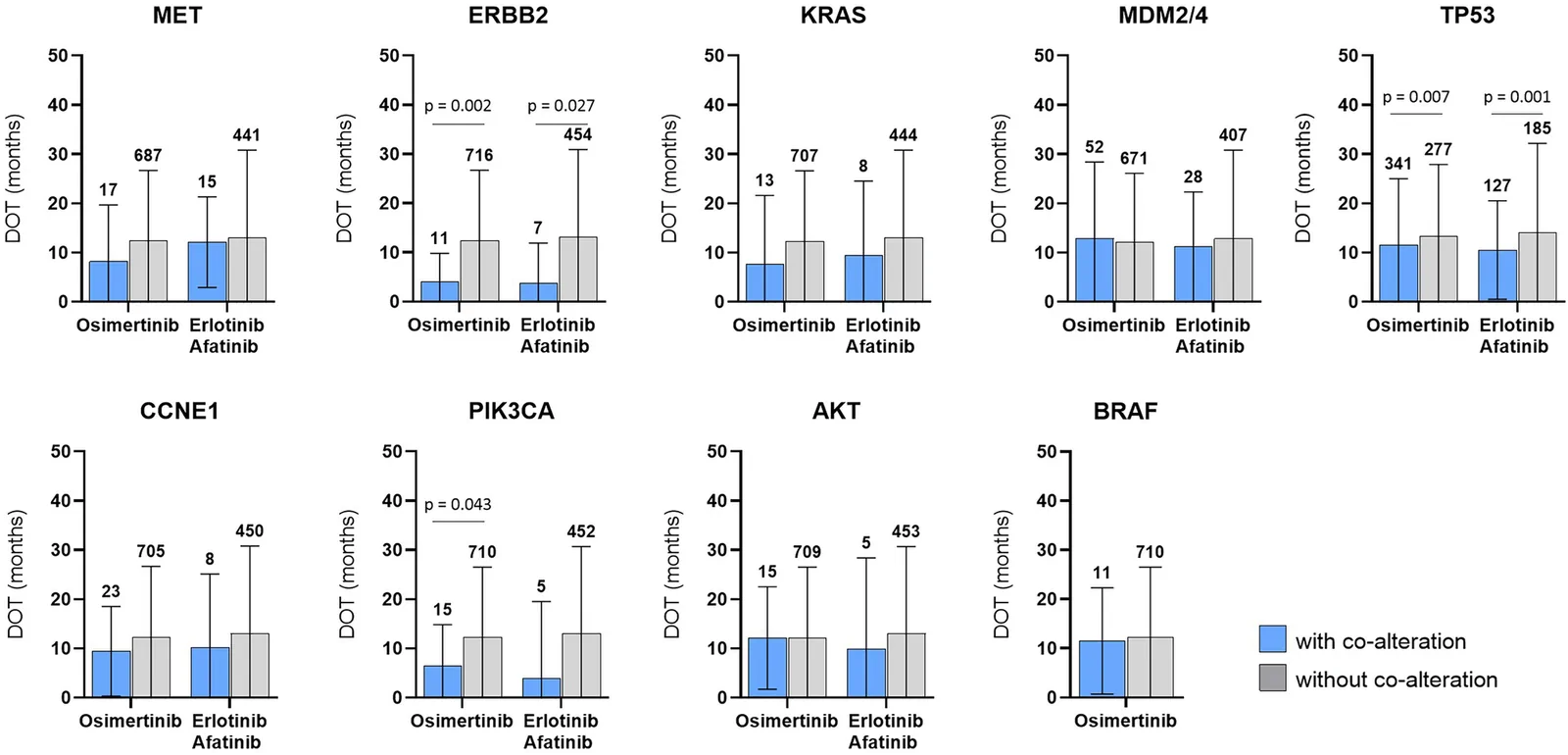
Duration of treatment in EGFR^+^ NSCLC in FH-FMI CGDB based on co-alteration status and first-line treatment with EGFRi. Median duration of treatment (DOT, months) with EGFRi (osimertinib and erlotinib/afatinib) according to the presence (blue) or absence (gray) of specific molecular co-alterations in the FH-FM-CGDB cohort. Numbers above each bar indicate the number of patients in the respective subgroup. Vertical lines represent the interquartile range (IQR). Statistical comparisons were performed using the Wilcoxon rank-sum test; *p* < 0.05 are shown.

**Fig. 4:**
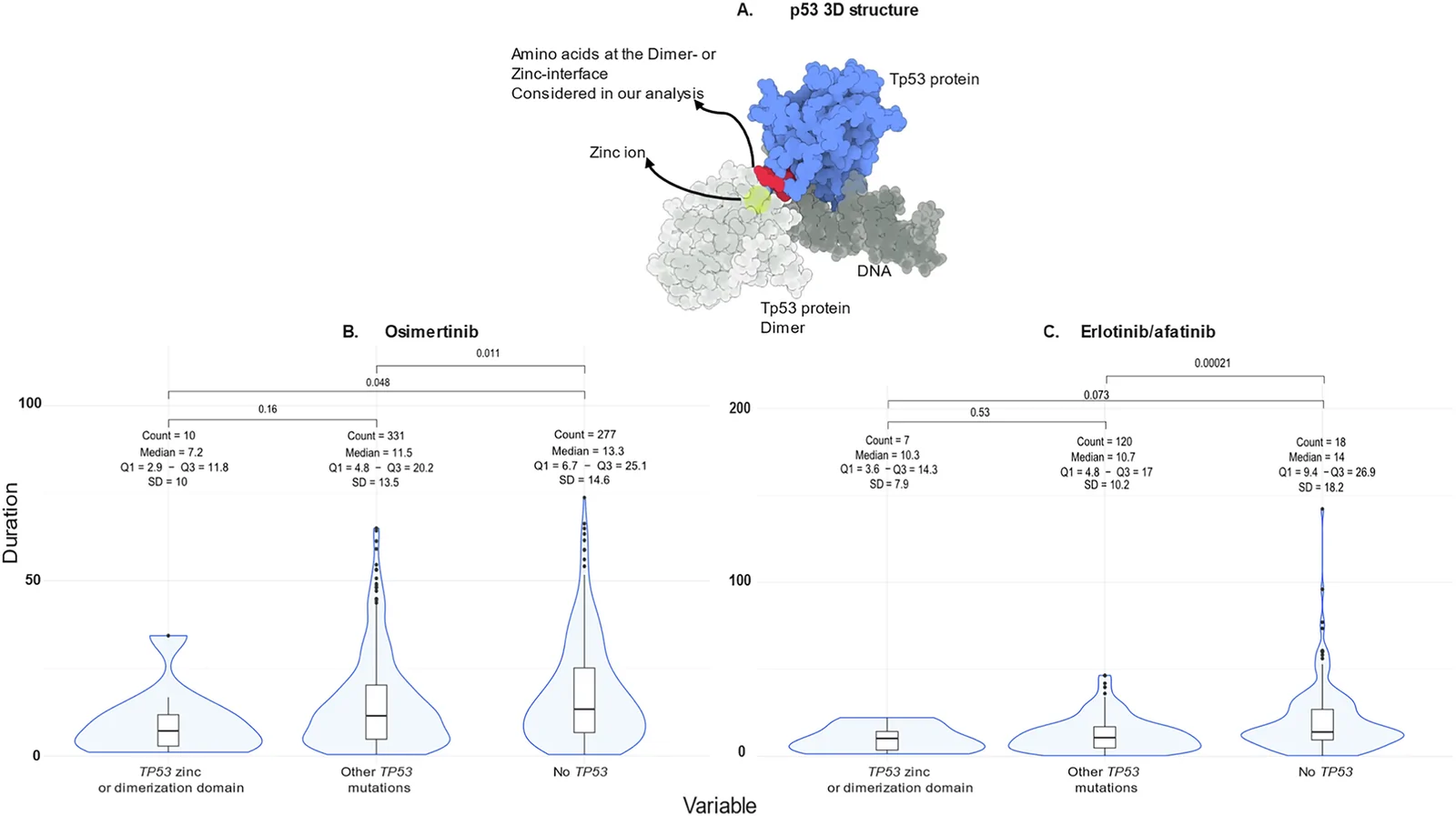
TP53 mutations association with duration of treatment on first-line EGFRi treatment within the EGFR^+^ NSCLC patients of FH-FMI-CGDB cohort. **A** Three-dimensional structure of the p53 tumor suppressor protein highlighting amino acids at the dimerization and zinc-binding interfaces included in our analysis. The DNA-binding domain (blue) interacts with DNA (dark gray), stabilized by a coordinated zinc ion (yellow). Residues at the zinc-binding interface are shown in red. The dimerization region (light gray) contributes to oligomerization and overall protein stability. **B** Duration of treatment (DOT, months) for first-line therapy with osimertinib according to TP53 status: mutations in the zinc-binding or dimerization domain, other TP53 mutations, or no TP53 mutations. **C** Duration of treatment (DOT, months) for first-line therapy with erlotinib/afatinib according to TP53 status: mutations in the zinc-binding or dimerization domain, other TP53 mutations, or no TP53 mutations. For each group, the number of patients (count), median DOT (median), interquartile range (IQR, Q1–Q3), and standard deviation (SD) are shown. Statistical comparisons between groups were performed using the Wilcoxon rank-sum test, and *p*-values are indicated.

In the FH-FMI CGDB cohort, 341 out of 618 patients (55%) with *EGFR*^+^ NSCLC treated with first-line osimertinib harbored a *TP53* alteration (any; 125 distinct *TP53* alterations were detected) (Fig. 3; Supplementary Table 2). These patients exhibited a significantly shorter DOT compared to those without TP53 alterations (median DOT 11.5 months (IQR 4,7–20 months) vs. 13.3 months (IQR 6.7–25.1 months); *p* = 0.0066). In the USZ cohort, a descriptive pattern in the same direction was observed (7 months (IQR 5–21 months) vs. 21 months (IQR 11–30 months); *p* = 0.1946) (Supplementary Table 3). A residue-based classification focusing on the *TP53* zinc-binding (176, 179, 238, 242) and dimerization interface (177, 178, 179, 181) amino acids revealed that alterations affecting these specific residues were associated with numerically shorter DOT on osimertinib when compared to cases with *TP53* mutations affecting other areas of the gene (Fig. 4).

A similar pattern was observed in both datasets: FH-FMI CGDB patients had a median DOT on osimertinib of 7.2 months (IQR 2.9–11.8 months) in cases with *TP53* mutations in the zinc-binding or dimerization domain vs. 11.5 months (IQR 4.8–20.2 months *p* = 0.16) in cases with other *TP53* mutations and vs 13.3 months (IQR 6.7–25.1 months, *p* = 0.048) in cases without *TP53* alterations (Fig. 4). In the USZ cohort, a similar descriptive pattern was observed, with a median DOT of 6.5 months (IQR not estimable) vs. 8 months (IQR 6–25 months). Due to the small sample size (*n* = 2 vs. *n* = 7), no formal statistical comparison was performed in the USZ cohort.

**Fig. 5:**
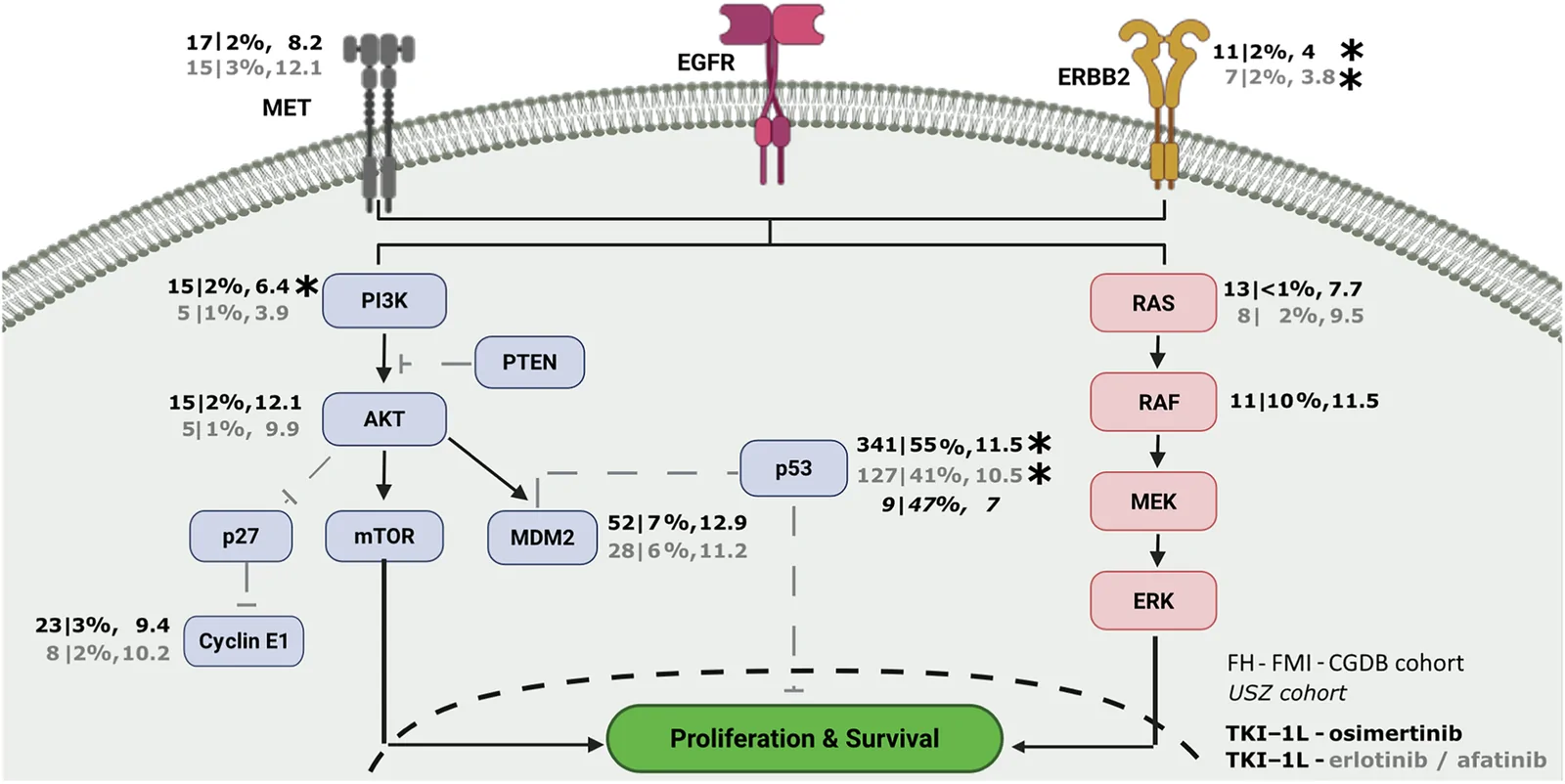
Landscape of the co-alterations within the downstream signaling pathways in EGFR^+^ NSCLC. The flow chart shows the key receptor tyrosine kinases and downstream signaling pathways implicated in resistance to EGFRi. The alterations are mapped onto the EGFR, PI3K/AKT/mTOR pathway (gray boxes) and RAS/RAF/MEK/ERK pathway (red boxes), cell-cycle regulators and p53 as central survival and proliferation pathways signaling cascades. Median of duration of treatment (DOT) on the first systemic treatment line is reported next to gene frequency (n), percentage (%), and median DOT (months) - with first-line EGFRi treatment osimertinib (black letters) or erlotinib/afatinib (gray letters). Roman writing designated FH-FMI CGDB cohort and italic USZ cohort (p53). Asterisks (*) indicate alterations with statistically significant associations to clinical outcomes. Flow chart created in Biorender https://BioRender.com.

Also, in the FH-FMI CGDB patients, who received the EGFRi afatinib/erlotinib in the first treatment line, DOT was significantly shorter for patients whose tumors were harboring a co-occurring *TP53* mutation (10.5 months (IQR 4.7–17.1 months) vs. 14 months (IQR 9.4–26.9 months); *p* < 0.01) (Supplementary Table 3, Fig. 5).

Using the residue-based functional classification of *TP53* mutations, no statistically significant difference was observed among FH-FMI CGDB patients treated with afatinib/erlotinib, with a median DOT of 10.3 months (IQR 3.6–14.3 months) in cases with *TP53* mutations in the zinc-binding or dimerization domain vs.10.7 months (IQR 4.8–17 months; *p* = 0.53) in cases with other *TP53* mutations and vs. 14 months (IQR 9.4–26.9; *p* = 0.073) in cases without *TP53* alterations (Fig. 4). No *EGFR*^+^ NSCLC patients with *TP53* co-alterations in the USZ cohort were treated with afatinib/erlotinib in the first-line setting (Supplementary Table 3).

### Association of other co-alterations with DOT in *EGFR*^+^ NSCLC on EGFRi in the first treatment line

Assessing other co-alterations, a significantly shorter DOT for treatment with the EGFRi osimertinib was also detected in *EGFR*^+^ NSCLC patients in the FH-FMI CGDB whose tumors were harboring a co-occurring *ERBB2 amplification* (4 months (IQR 2.4–6.4 months) vs. 12.4 months (IQR 5.8–22.9 months); *p* < 0.0019) or *PIK3CA* mutation (6.4 months (3.3–11.7 months) vs. 12.3 months (IQR 5.7–22.7 months); *p* = 0.043) (Fig. 5**;** Supplementary Table 3). A numerical trend towards a shorter DOT was also detected for cases with co-occurring *MET* and *CCNE1* amplifications and mutations in *KRAS* and *BRAF* (Fig. 3; Fig. 5, Supplementary Table 3). The DOT on EGFRi afatinib/erlotinib was significantly shorter for *EGFR*^+^ NSCLC patients in the FH-FMI CGDB whose tumors were harboring a co-occurring amplification in *ERBB2* (3.8 months (IQR 2.6–9.5 months) vs. 13.1 months (IQR 8.1–22.6 months); *p* = 0.03) or a mutation in *TP53* (see above) (Supplementary Table 3). A trend towards a shorter DOT was also recorded for cases with co-occurring *MDM2/4* amplification, *MET* amplifications or mutations *in KRAS, PIK3CA* and *CCNE1* (Fig. 5, Supplementary Table 3). Assessment of the DOT for osimertinib and afatinib/erlotinib treatment in the USZ cohort in the context of co-alterations next to TP53 was limited due to small patient numbers in the respective subgroups. Assessing the USZ cohort, a strong positive correlation between DOT and PFS was observed (Spearman rs = 0.74; *P* < 0.05, *n* = 42) (Supplementary Fig 3).

## Discussion

In this analysis, conducted on two complementary RWD cohorts in the POP framework, we describe the co-alteration landscape of advanced *EGFR*^+^ NSCLC and explore associations between these co-alterations and DOT on first-line treatment with EGFRi. With the recently introduced new first-line treatment options for *EGFR*^+^ NSCLC, understanding and anticipating EGFRi resistance becomes crucial^9,10,21^ to inform future strategies for treatment escalation and de-escalation in a personalized oncology approach based on individual clinicogenomic factors. Within this context, our findings provide hypothesis-generating evidence that may support more refined, biology-driven treatment stratification. Analysing the co-alteration landscape in both cohorts (USZ and FH-FMI CGDB) revealed complex molecular profiles, harboring at least one co-alteration associated with *EGFR* signaling with co-alterations in *TP53* being most frequent. This high percentage of co-alterations highlights the potential insight gained by expanding our understanding of co-alteration patterns and how they impact treatment outcomes.

TP53 mutations were associated with a shorter DOT on osimertinib in the FH-FMI CGDB cohort. In the USZ cohort, a similar descriptive pattern was observed.

In the literature, not all *TP53* mutations were reported to have the same impact^24^. Especially, mutations in exon 5-8, as well as disruptive mutations, are known for reduced progression-free survival (PFS) in advanced *EGFR*^+^ NSCLC treated with EGFRi^2,25^. Our functional amino acid level-based analysis suggests that *TP53* mutations affecting the zinc-binding or tetramerization domains are associated with shorter DOT on first-line osimertinib and afatinib/erlotinib compared to cases with other *TP53* mutations and without *TP53* mutations. Given the exploratory nature of the residue-level analyses and the number of comparisons performed, these results generate hypotheses for further investigation in dedicated studies.

These types of alterations typically cause profound structural disruption of the p53 protein, resulting in complete loss of tumor suppressor function. The zinc-binding region stabilizes the DNA-binding domain by coordinating a zinc ion through conserved cysteine and histidine residues. The disruption of this region destabilizes the DNA-binding domain, leading to structural collapse and complete loss of DNA-binding capacity^26,27^. Similarly, mutations in the dimerization domain of p53 impair the ability of the protein to form its physiologically active tetrameric complex, which is essential for its tumor suppressor function, thereby resulting in loss of transcriptional activity and dominant-negative effects^28,29^. In contrast, mutations distributed more broadly across the DNA-binding domain do not uniformly produce such dramatic structural consequences. Many of these are contact mutations that selectively impair DNA interaction while preserving overall protein folding, allowing partial residual function^26,30^. This biological distinction likely explains why disruptive structural mutations—particularly those affecting zinc coordination or tetramerization—are associated with poorer clinical outcomes, whereas contact mutations appear less impactful.

From a therapeutic perspective, our findings highlight the potential clinical relevance of integrating domain-specific *TP53* alteration assessment in clinical decision-making in advanced *EGFR*^+^ NSCLC as early as the first treatment line. Incorporating detailed mutation profiling—beyond the mere presence or absence of *TP53* alterations—may contribute to identifying patients for escalation/de-escalation treatment strategies and could become crucial to further inform choices within the standard-of-care options.

Furthermore, in the FH-FMI CGDB cohort, co-alterations such as *ERBB2* and *MET* amplifications, and mutations in *PIK3CA, KRAS* and *BRAF* were associated with reduced DOT on osimertinib. Notably, most of these alterations are targetable with established therapies in advanced NSCLC. However, data on the co-occurrence of EGFR exon 19 deletions or L858R mutations with additional oncogenic alterations - upfront and their impact on treatment outcome with osimertinib monotherapy versus combined regimens, such as those used in FLAURA 2 or MARIPOSA - remain limited. Our analysis showed that these co-alterations were associated with shorter DOT on first-line EGFRi in advanced *EGFR*^+^ NSCLC. This suggests that co-alterations may be relevant for treatment stratification; early in the treatment course. However, this requires validation in prospective clinical trials and/or real-world studies.

Co-alterations in our *EGFR*^+^ NSCLC datasets may reflect either shared clonal events or subclonal heterogeneity across tumor cell populations. Based on routine clinical procedure in our dastasets standard of care molecular testing at a single time point was available per patient, precluding assessment of clonal architecture or evolutionary dynamics. Within these constraints, our analysis captures consistent, biologically plausible associations of specific co-alterations with shorter DOT, providing potentially clinically relevant insights in a real-world setting and generating a hypothesis for future studies exploring the underlying biological and translational mechanisms.

The conventional gold standard for treatment recommendations–hypothesis-driven, prospective randomized clinical trials–becomes increasingly impractical in assessing the variety of resistance mechanisms to specific anti-cancer drugs and their impact on DOT promptly. Therefore, the efficient use of the RWD could bridge the gap between the growing number of molecular alterations and the need for data-driven decision-making in precision oncology. Our study represents a comprehensive approach combining a broad, representative analysis of clinically relevant co-alterations and their impact on DOT in over 20,000 advanced NSCLC cases from the clinicogenomic database FH-FMI CGDB^31^ with an in-depth examination of mutational patterns and clinical treatment courses in the POP USZ cohort.

Early-generation EGFRi, such as erlotinib and afatinib, no longer play a central role in the rapidly evolving first-line treatment landscape for advanced *EGFR*^+^ NSCLC. However, they are used in later-line settings and cases involving uncommon *EGFR* mutations. Their role in the current treatment sequence, particularly in the context of resistance mechanisms, remains to be fully defined. Our analysis provides insights into DOT on these early-generation EGFRi in the presence of specific co-alterations, highlighting that certain alterations – particularly *ERBB2* amplifications and *TP53* mutations - are associated with shorter DOT on these agents.

Treatment histories differed between the two cohorts, reflecting the different timeframes of SACT administration and evolving treatment practices. The FH-FMI CGDB cohort spans a SACT period of twenty-two years from up to 280 US cancer clinics, also covering an era when chemotherapy was the first-line SACT in advanced *EGFR*^+^ NSCLC. To integrate the profound change of treatment regimens employed and the improved clinical management, we assessed the time of treatment initiation in the FH-FMI CGDB cohort, which concentrated in more recent years, reflecting evolving standards of care. While this supports a certain degree of comparability with contemporary clinical practice, temporal changes in treatment strategies may still have influenced DOT. In contrast, the USZ cohort reflects more recent treatment patterns in Switzerland. This illustrates how complementary RWD cohorts can reveal chronological and geographical differences in clinical practice, emphasizing the importance of context in data interpretation.

RWD are mostly derived from EHRs, which capture clinical and molecular data from clinical routines across multiple sites, rather than the specific data and examination results collected in clinical trials. As a result, in the context of RWD, missing data is common and must be considered when interpreting findings. Although the advanced NSCLC cohort in the FH-FMI CGDB is significantly larger than most available datasets, sub-grouped cohort sizes shrink considerably when multiple criteria are applied, limiting statistical power (e.g., advanced NSCLC, specific *EGFR* mutation, treatment line, systemic treatment employed or specific co-alterations). This underscores the importance of collaborative efforts to ensure sufficiently large cohort sizes for meaningful analysis.

The analysis presented is based on a singular molecular assessment before starting the first-line SACT, which limits the informative value to up-front resistances. Assessing the development of resistance mechanisms requires a longitudinal approach, capturing resistance alterations as they progress during sequential treatment lines in individual patients. To create comparable and reliable longitudinal results, molecular testing should be conducted before and after each treatment line, an approach that is not necessarily reflected in RWD.

Molecular assessments were conducted both on tissue biopsies and liquid biopsies. Liquid biopsies provide a less invasive approach and are particularly valuable for patients with tumors not amenable to tissue biopsy sampling. They are increasingly integrated into clinical practice, yet their sensitivity is lower and false-negative results may occur. In this context, the use of liquid biopsy may have contributed to an underestimation of co-alterations in *EGFR*^+^ NSCLC cases evaluated exclusively by liquid biopsy. Given the limited size of this subgroup, however, a substantial impact on the overall findings appears unlikely.

A very important limitation of this study is the lack of more robust outcome parameters for EGFRi therapy. The CGDB database captures duration of therapy but not endpoints such as objective response rate or progression-free survival (PFS), both based on RECIST evaluation of tumors. DOT is frequently used as a pragmatic endpoint in RWD analyses, particularly when structured radiological assessments required for PFS are not consistently available. However, DOT reflects a composite endpoint influenced not only by disease control but also by treatment tolerability and clinical decision-making. Although previous analyses have demonstrated a correlation between DOT and PFS at the patient level across multiple treatment classes in oncology, this varies depending on treatment modality, and DOT has not been validated as a surrogate endpoint for PFS or overall survival^32^.

To address this limitation, we explored the relationship between DOT and PFS in the USZ cohort. DOT showed a strong positive correlation with PFS, supporting its use as a pragmatic outcome measure in our analyses. However, due to the limited sample size and the, thus, purely descriptive nature of this additional assessment, the findings cannot be generalized to the EGFR^+^ NSCLC population as a whole.

A further important limitation is the absence of multivariable analyses to account for potential confounding factors. While variables such as performance status, tumor burden, and comorbidities may influence treatment duration, these were not consistently available or linkable at the individual patient level in the partly aggregated dataset used for this study. As a result, the analyses presented are univariate and present exploratory associations.

Despite these limitations, this study provides insight into the co-alteration landscape of advanced *EGFR*^+^ NSCLC in two complementary real-world datasets. The findings are hypothesis-generating and support further investigation of co-alterations, particularly TP53, as potential biomarkers in prospective studies.

Furthermore, this work illustrates how RWD can complement traditional clinical research by capturing molecular and clinical heterogeneity in routine practice and by enabling the exploration of clinically relevant associations that may not be readily accessible in randomized trials. In this context, RWD may contribute to a more nuanced understanding of treatment outcomes and support clinical interpretation in complex decision-making scenarios.

Future studies are required to determine whether the reported co-alterations associated with shortened DOT can be translated into actionable strategies and, thus, can ultimately inform treatment selection in *EGFR*^+^ NSCLC in clinical practice.

## Methods

### Ethics statement and consent to participate

This study was conducted in accordance with the principles of the Declaration of Helsinki. The protocol was approved by the Cantonal Ethics Committee Zurich, Switzerland (KEK Zürich; Project ID: 2022-02289) and is registered at ClinicalTrials.gov (NCT06680726). Written informed consent was obtained from all participants before inclusion. Deceased patients were included only where a signed general consent permitting the use of data was available. All data were pseudonymised to ensure patient confidentiality.

### Cohort assembly and data collection

Cohort assembly and data collection were conducted within the POP framework, which has been previously described^22^. Patients with advanced *EGFR*^+^ NSCLC meeting the eligibility criteria were identified and recruited at USZ between 12/2023 and 12/2024 with included patient histories dating back to October 2014 (ethics approval: BASEC ID 2022-02289, NCT06680726). *EGFR*^+^ was defined as a cancer harboring an *EGFR* exon 19 deletion and/or an *EGFR* L858R mutation. Within the POP framework, this cohort was matched to a patient cohort from the FH-FMI NSCLC CGDB using a bespoke predefined matching algorithm as previously described^22^. In brief, matching was based on a predefined set of variables containing relevant clinical, immunohistochemical and molecular features to identify de-identified patients with comparable clinicogenomic profiles in the FH-FMI-CGDB. Based on this matching, aggregated cohort-level clinical, molecular, and outcome data were provided for analysis. The matching strategy was developed to balance clinically meaningful similarity with a cohort size sufficient for analysis.

Clinicogenomic data for USZ patients were collected from the USZ internal electronic health records (EHR). The clinicogenomic data for the FH-FMI CGDB cohort were extracted from the FH-FMI CGDB database, incorporating de-identified data from up to 280 US cancer clinics with linked genomic data derived from Foundation Medicine’s comprehensive genomic profiling tests as previously described^22^. Data was collected between January 2011 and March 2024 with included patient histories dating back to January 2002. Sub-cohorts were comprised according to co-alteration profiles and the EGFRi in the USZ and the CGDB cohort (Fig. 1). Sub-cohorts with six or fewer CGDB patients were excluded from reporting following the FH-FMI CGDB data protection guideline.

No formal sample size calculation was performed; cohort size was defined by data availability in the respective real-world datasets.

### Molecular testing and alterations of interest

Molecular testing was conducted before or immediately at the start of the first-line systemic anti-cancer therapy (SACT) with an EGFRi across both cohorts within the clinical standard of care. FoundationOne*®* CDx and FoundationOne*®* CDx Liquid were used as diagnostic tests at USZ and in the cancer centers whose patients‘ EHRs were collected in the CGDB^33^. Alterations of interest for this analysis were defined as alterations of elements of *EGFR* signaling and alternative routes of activation of downstream signaling such as *MET* amplification, *ERBB2* amplification, *ERBB2* exon 20 insertion mutation, *KRAS* mutation, *BRAF-V600E* mutation, *PIK3CA* hotspot mutation, *AKT* amplification, *PTEN* mutation, *MDM 2/4* amplification, *TP53* mutation, *CCNE1* amplification. Alterations were defined as previously described and were detected according to the standard of care assessment guidelines for the diagnostic molecular tests used at the specific institution^34^. *TP53* alterations were further stratified based on the localization within the specific functional domains of the p53 protein, namely the zinc-binding domain (amino acids 176 – 239), the DNA-binding domain (amino acids 102 – 292), and the tetramerization (dimerization) domain (amino acids 323 – 356)^30^. *TMB* and *PD-L1* levels were determined by local testing and reported according to standardized cutoffs.

### Statistical analysis

Duration of treatment (DOT) was used as the key outcome parameter. DOT on first-line EGFRi was compared between subgroups of patients with and without co-alterations in the FH-FMI CGDB cohort. Analyses in the USZ cohort were descriptive in nature. Descriptive methods were used to summarize and present the data. The continuous variables were reported as medians with interquartile ranges (IQR) to account for non-normally distributed data. Confidence intervals for median-based comparisons were not calculated, as individual-level data were not consistently available in the aggregated dataset. The categorical variables were presented as frequencies and percentages. To assess the differences between the groups in the FH-FMI CGDB cohort, we used a Wilcoxon rank-sum test as the samples were not normally distributed and groups were independent. No formal statistical comparisons were performed in the USZ cohort due to the limited sample size. *P* < 0.05 were considered statistically significant. *P*-values were reported without adjustment for multiple testing, as analyses were exploratory and hypothesis-generating. Statistical analyses were conducted using R version 4.3.1^35^. Missing data were not imputed, and analyses were performed on available data. A descriptive correlation between DOT and progression-free survival (PFS) was assessed in the USZ cohort using Spearman’s rank correlation coefficient. Patients without an observed PFS event at last follow-up were excluded from this analysis. To account for temporal changes in treatment practices, the distribution of treatment initiation over time was descriptively assessed in the FH-FMI CGDB cohort.

## Supplementary information

Supplementary Information

## Data Availability

All data supporting the findings of this study are available within the paper and its Supplementary Information. The data that support the findings of this study are available from Foundation Medicine, F. Hoffmann-La Roche AG and University Hospital Zürich but restrictions apply to the availability of these data, which were used under licence for the current study, and so are not publicly available. Some data, however, could be available from the authors upon reasonable request and with permission of Foundation Medicine, F. Hoffmann-La Roche AG, Genentech Inc. and University Hospital Zürich.

## References

[1] Lynch, T. J. et al. Activating mutations in the epidermal growth factor receptor underlying responsiveness of non-small-cell lung cancer to gefitinib. *N. Engl. J. Med.* **350**, 2129–2139 (2004). 10.1056/NEJMoa040938 15118073

[2] Molina-Vila, M. A. et al. Nondisruptive p53 mutations are associated with shorter survival in patients with advanced non-small cell lung cancer. *Clin. Cancer Res.* **20**, 4647–4659 (2014). 10.1158/1078-0432.CCR-13-2391 24696321

[3] Zhang, T. et al. Treatment of uncommon EGFR mutations in non-small cell lung cancer: new evidence and treatment. *Transl. Lung Cancer Res.* **8**, 302–316 (2019). 10.21037/tlcr.2019.04.12 PMC6626855 31367543

[4] Passaro, A. et al. ESMO expert consensus statements on the management of <em>EGFR < /em> mutant non-small-cell lung cancer. *Ann. Oncol.* **33**, 466–487 (2022). 10.1016/j.annonc.2022.02.003 35176458

[5] Ramalingam, S. S. et al. Overall survival with Osimertinib in untreated, EGFR-mutated advanced NSCLC. *N. Engl. J. Med.* **382**, 41–50 (2020). 10.1056/NEJMoa1913662 31751012

[6] Leonetti, A. et al. Resistance mechanisms to osimertinib in EGFR-mutated non-small cell lung cancer. *Br. J. Cancer* **121**, 725–737 (2019). 10.1038/s41416-019-0573-8 PMC6889286 31564718

[7] Passaro, A., Jänne, P. A., Mok, T. & Peters, S. Overcoming therapy resistance in EGFR-mutant lung cancer. *Nat. Cancer* **2**, 377–391 (2021). 10.1038/s43018-021-00195-8 35122001

[8] Stewart, E. L., Tan, S. Z., Liu, G. & Tsao, M. S. Known and putative mechanisms of resistance to EGFR targeted therapies in NSCLC patients with EGFR mutations-a review. *Transl. Lung Cancer Res.* **4**, 67–81 (2015). 10.3978/j.issn.2218-6751.2014.11.06 PMC4367712 25806347

[9] Planchard, D. et al. Osimertinib with or without chemotherapy in EGFR-mutated advanced NSCLC. *N. Engl. J. Med.* **389**, 1935–1948 (2023). 10.1056/NEJMoa2306434 37937763

[10] Popat, S. et al. LBA54 Amivantamab plus chemotherapy vs chemotherapy in EGFR-mutated, advanced non-small cell lung cancer after disease progression on osimertinib: Second interim overall survival from MARIPOSA-2. *Ann. Oncol.* **35**, S1244–S1245 (2024).

[11] Brown, C. J., Lain, S., Verma, C. S., Fersht, A. R. & Lane, D. P. Awakening guardian angels: drugging the p53 pathway. *Nat. Rev. Cancer* **9**, 862–873 (2009). 10.1038/nrc2763 19935675

[12] Kandoth, C. et al. Mutational landscape and significance across 12 major cancer types. *Nature* **502**, 333–339 (2013). 10.1038/nature12634 PMC3927368 24132290

[13] Blanden, A. R. et al. Zinc shapes the folding landscape of p53 and establishes a pathway for reactivating structurally diverse cancer mutants. *Elife* **9**, 10.7554/eLife.61487 (2020). PMC7728444 33263541

[14] Ha, J.-H., Prela, O., Carpizo, D. R. & Loh, S. N. p53 and Zinc: a malleable relationship. *Front. Mol. Biosci.* **ume 9**, 2022 (2022). 10.3389/fmolb.2022.895887 PMC9043292 35495631

[15] Attili, I., Fabrizio, F. P. & Marinis, F. Co-occurring genomic alterations in NSCLC: making order into a crowded list. *Cancers* **17**, 10.3390/cancers17142388 (2025). PMC12293187 40723270

[16] Canale, M. et al. The role of TP53 mutations in EGFR-mutated non-small-cell lung cancer: clinical significance and implications for therapy. *Cancers* **14**, 10.3390/cancers14051143 (2022). PMC8909869 35267450

[17] Ibusuki, R. et al. TP53 gain-of-function mutations promote osimertinib resistance via TNF-α-NF-κB signaling in EGFR-mutated lung cancer. *NPJ Precis Oncol.* **8**, 60 (2024). 10.1038/s41698-024-00557-2 PMC10908812 38431700

[18] Lan, B., Zhao, N., Du, K. & Leng, B. Concurrent TP53 mutations predict a poor prognosis of EGFR-mutant NSCLCs treated with TKIs: An updated systematic review and meta-analysis. *Oncol. Lett.* **24**, 384 (2022). 10.3892/ol.2022.13504 PMC9494618 36238360

[19] Sun, H. et al. Optimal therapy for concomitant EGFR and TP53 mutated non-small cell lung cancer: a real-world study. *BMC Cancer* **23**, 198 (2023). 10.1186/s12885-023-10637-4 PMC9979422 36864384

[20] Viray, H. et al. A real-world study of patient characteristics and clinical outcomes in egfr mutated lung cancer treated with first-line Osimertinib: expanding the FLAURA trial results into routine clinical practice. *Cancers* **16**, 10.3390/cancers16061079 (2024). PMC10968877 38539415

[21] Passaro, A. et al. 1254MO Safety and anti-tumour activity of zipalertinib in NSCLC patients (pts) with EGFR exon 20 insertion (ex20ins) mutations who received prior amivantamab. *Ann. Oncol.* **35**, S803–S804 (2024).

[22] Boos, L. A. et al. Precision Oncology Program (POP), an observational study using real-world data and imaging mass cytometry to explore decision support for the Molecular Tumor Board: study protocol. *BMJ Open* **15**, e096591 (2025). 10.1136/bmjopen-2024-096591 PMC11950961 40139698

[23] Flatiron. Flatiron Health. Database Characterization, https://flatiron.com/database-characterization (2025).

[24] Hobor, S. et al. Mixed responses to targeted therapy driven by chromosomal instability through p53 dysfunction and genome doubling. *Nat. Commun.* **15**, 4871 (2024). 10.1038/s41467-024-47606-9 PMC11176322 38871738

[25] Coleman, N. et al. Beyond epidermal growth factor receptor: MET amplification as a general resistance driver to targeted therapy in oncogene-driven non-small-cell lung cancer. *ESMO Open* **6**, 100319 (2021). 10.1016/j.esmoop.2021.100319 PMC8637467 34837746

[26] Joerger, A. C. & Fersht, A. R. Structural biology of the tumor suppressor p53. *Annu. Rev. Biochem.* **77**, 557–582 (2008). 10.1146/annurev.biochem.77.060806.091238 18410249

[27] Vousden, K. H. & Lane, D. P. p53 in health and disease. *Nat. Rev. Mol. Cell Biol.* **8**, 275–283 (2007). 10.1038/nrm2147 17380161

[28] Kamada, R., Nomura, T., Anderson, C. W. & Sakaguchi, K. Cancer-associated p53 tetramerization domain mutants: quantitative analysis reveals a low threshold for tumor suppressor inactivation. *J. Biol. Chem.* **286**, 252–258 (2011). 10.1074/jbc.M110.174698 PMC3012982 20978130

[29] Clore, G. M. et al. High-resolution structure of the oligomerization domain of p53 by multidimensional NMR. *Science* **265**, 386–391 (1994). 10.1126/science.8023159 8023159

[30] Cho, Y., Gorina, S., Jeffrey, P. D. & Pavletich, N. P. Crystal structure of a p53 tumor suppressor-DNA complex: understanding tumorigenic mutations. *Science* **265**, 346–355 (1994). 10.1126/science.8023157 8023157

[31] Liu, R. et al. Systematic pan-cancer analysis of mutation-treatment interactions using large real-world clinicogenomics data. *Nat. Med.* **28**, 1656–1661 (2022). 10.1038/s41591-022-01873-5 35773542

[32] Blumenthal, G. M. et al. Analysis of time-to-treatment discontinuation of targeted therapy, immunotherapy, and chemotherapy in clinical trials of patients with non-small-cell lung cancer. *Ann. Oncol.* **30**, 830–838 (2019). 10.1093/annonc/mdz060 30796424

[33] Frampton, G. M. et al. Development and validation of a clinical cancer genomic profiling test based on massively parallel DNA sequencing. *Nat. Biotechnol.* **31**, 1023–1031 (2013). 10.1038/nbt.2696 PMC5710001 24142049

[34] Medicine, F. *FoundationOne®CDx*, https://www.foundationmedicine.com/test/foundationone-cdx (2026).

[35] The R Project for Statistical Computing. A language and environment for statistical computing (2023).

