# Supplementary material for: Co-alterations associated with resistance to EGFR inhibitors in advanced EGFR-positive NSCLC: insights from the precision oncology program in two large international real-world cohorts
Source: NPJ Precis Oncol. 2026 Jul 22;10:436. doi: 10.1038/s41698-026-01617-5 (PMC13755411; doi:10.1038/s41698-026-01617-5)
Supplement: Supplementary file 1 [file 41698_2026_1617_MOESM1_ESM.pdf]

Supplementary Information

Co-Alterations Associated with Resistance to EGFR Inhibitors in advanced EGFR-positive NSCLC: Insights from the Precision Oncology Program in Two Large International Real-World Cohorts.

Boos LA, Doerig C, Camarillo-Retamosa E, Sarwary M, Rizzo S, Gut G, Miglino N, Fábregas-Ibáñez L, Früchtenicht C, Zoche M, Bodenmiller B, Chevrier S, Eklund AS, Nowak M, Rahmani-Khajouei S, Berardo GC, Kaczmarek L, Bosshard K, Archey W, Bodmer M, Glinz D, Opitz I, Ulrich S, Guckenberger M, Hempel L, Stiefel I, Rahimzadeh P, Gosztonyi B, Richter U, Bankel L, Wicki A

Supplementary Table 1: First-line systemic anti-cancer treatments administered in the study cohorts. The table contains the numbers and proportion of treated patients for each regimen (n (%)) with the median of the duration of the treatment (DOT) (months from treatment initiation to discontinuation for any reason, including progression or toxicity) and the interquartile range (IQR). Dash (-) indicates treatments with missing or unevaluable DOT data.

		FH-FMI-CGDB	median DOT FH-FMI-CGDB	USZ	median DOT USZ
**First Line Treatment**		**N = 1927**	**months (IQR)**	**N = 43**	**months (IQR)**
**EGFRi**	Afatanib	122 (6.3 %)	11.4 (6.3 - 19.7)	1 (2.3%)	8 (-)
	Erlotinib	346 (17.9%)	13.4 (8.2 - 23.0)	4 (9.3%)	36.5 (27 - 37)
	Gefitinib	13 (0.7%)	12.2 (8.2 - 20.2)	-	-
	Osimertinib	730 (37.9%)	12.2 (5.7 - 22.7)	22 (51.2%)	16.5 (7 - 24)
**Immunotherapy (monotherapy)**	Pembrolizumab	17 (0.9%)	2.3 (1.5 - 3.1)	1 (2.3%)	25 (-)
**Chemotherapy (monotherapy)**	Pemetrexed	12 (0.6%)	4.3 (2.8 - 8.9)	-	-
**Combination therapy**	Carboplatin Pemetrexed Pembrolizumab	58 (3.0%)	2.3 (1.3 - 6.7)	6 (14%)	2.5 (2 - 3)
	Carboplatin Paclitaxel Bevacizumab	10 (0.5%)	4.8 (4.1 - 7.3)	-	-
	Carboplatin Pemetrexed Bevacizumab	28 (1.5%)	3.7 (2.9 - 7.8)	-	-
	Carboplatin Paclitaxel	45 (2.3%)	4.3 (2.3 - 13.5)	-	-
	Carboplatin Pemetrexed	65 (3.4%)	3.1 (2.0 - 8.2)	3 (7%)	1 (-)
	Cisplatin Pemetrexed	15 (0.8%)	6.3 (3.7 - 15.8)	1 (2.3%)	3 (-)
**Others**		466 (24.2%)		5 (11.6%)	14 (7 - 24)

Supplementary Figure 1. Distribution of treatment initiation in EGFR+ NSCLC over time in the FH-FMI CGDB cohort. The bar graph illustrates the temporal distribution of treatment initiation (from 2007 through 30 September 2023) across the dataset.

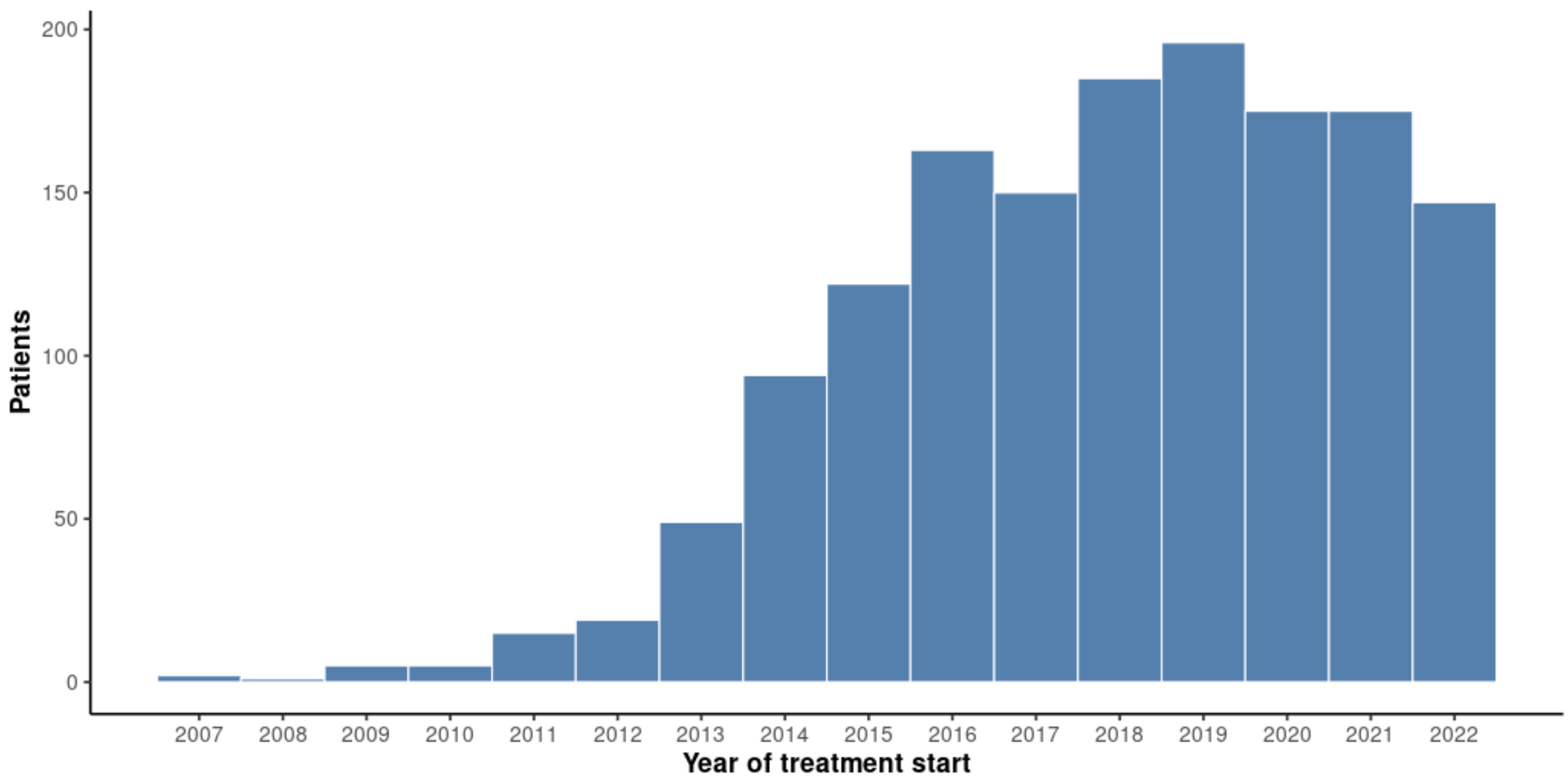

Supplementary Figure 2. Co-occurrence patterns of genomic alterations in advanced EGFR+ NSCLC across the study cohorts. The UpSet plots illustrate the frequency and distribution of co-occurring alterations in selected cancer-related genes in USZ **(a)** and FH-FMI-CGDB **(b)** cases. Vertical bars represent the number of cases harboring each specific combination of alterations, while the connected dots below indicate the genes involved. The numbers on the bars are the number of cases per co-occurring alteration.

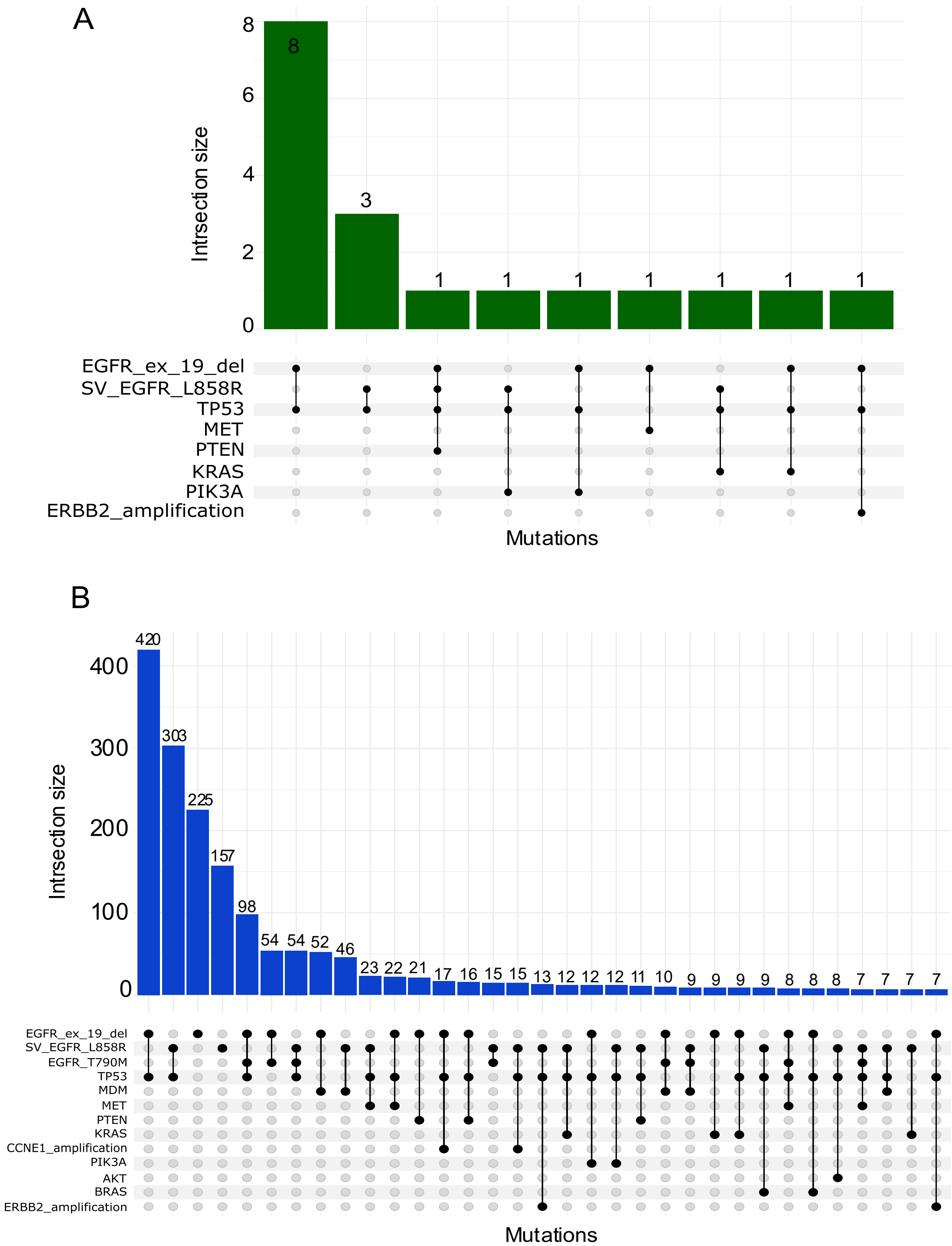

Supplementary Table 2: TP53 alterations detected in the FH-FMI-CGDB cohort. The table lists TP53 alterations identified in the FH-FMI-CGDB cohort. Each alteration contains amino acid changes, nucleotide changes, functional domain (zinc-binding amino acids 176–239, DNA-binding amino acids 102–292, tetramerization amino acids 323–356) and mutation type (missense, nonsense, frameshift, splice-site, copy number alteration).

Amino Acid Change	Nucleotide Change	Domain	Mutation Type
A138V		DNA-binding	Missense mutation
A159P		DNA-binding	Missense mutation
A159V		DNA-binding	Missense mutation
C135F		DNA-binding	Missense mutation
C135Y		DNA-binding	Missense mutation
C242F		DNA-binding	Missense mutation
C275F		DNA-binding	Missense mutation
C275Y		DNA-binding	Missense mutation
C277F		DNA-binding	Missense mutation
D259Y		DNA-binding	Missense mutation
D281Y		DNA-binding	Missense mutation
E224D		DNA-binding	Missense mutation
E285K		DNA-binding	Missense mutation
E286K		DNA-binding	Missense mutation
G105C		DNA-binding	Missense mutation
G105V		DNA-binding	Missense mutation
G154V		DNA-binding	Missense mutation
G244C		DNA-binding	Missense mutation
G244D		DNA-binding	Missense mutation
G245C		DNA-binding	Missense mutation
G245D		DNA-binding	Missense mutation
G245S		DNA-binding	Missense mutation
G245V		DNA-binding	Missense mutation
G262V		DNA-binding	Missense mutation
G266E		DNA-binding	Missense mutation
G266R		DNA-binding	Missense mutation
G266V		DNA-binding	Missense mutation
H193L		DNA-binding	Missense mutation
H193R		DNA-binding	Missense mutation
H193Y		DNA-binding	Missense mutation
H214R		DNA-binding	Missense mutation
I195T		DNA-binding	Missense mutation
K132N		DNA-binding	Missense mutation
K132R		DNA-binding	Missense mutation
L194R		DNA-binding	Missense mutation
M237I		DNA-binding	Missense mutation
M246V		DNA-binding	Missense mutation
N239D		DNA-binding	Missense mutation
P151S		DNA-binding	Missense mutation
P190L		DNA-binding	Missense mutation
P278L		DNA-binding	Missense mutation
P278R		DNA-binding	Missense mutation
P278S		DNA-binding	Missense mutation
R110L		DNA-binding	Missense mutation
R156P		DNA-binding	Missense mutation
R158H		DNA-binding	Missense mutation
R158L		DNA-binding	Missense mutation
R158P		DNA-binding	Missense mutation
R175G		DNA-binding	Missense mutation
R175H		DNA-binding	Missense mutation
R175L		DNA-binding	Missense mutation
R181P		DNA-binding	Missense mutation
R213L		DNA-binding	Missense mutation
R248L		DNA-binding	Missense mutation
R248Q		DNA-binding	Missense mutation
R248W		DNA-binding	Missense mutation
R249M		DNA-binding	Missense mutation
R249S		DNA-binding	Missense mutation
R267P		DNA-binding	Missense mutation
R273C		DNA-binding	Missense mutation
R273H		DNA-binding	Missense mutation
R273L		DNA-binding	Missense mutation
R273P		DNA-binding	Missense mutation
R280G		DNA-binding	Missense mutation
R280I		DNA-binding	Missense mutation

R280K		DNA-binding	Missense mutation
R280T		DNA-binding	Missense mutation
R282W		DNA-binding	Missense mutation
R283P		DNA-binding	Missense mutation
S127F		DNA-binding	Missense mutation
S215I		DNA-binding	Missense mutation
S241C		DNA-binding	Missense mutation
S241F		DNA-binding	Missense mutation
T155P		DNA-binding	Missense mutation
V143M		DNA-binding	Missense mutation
V157F		DNA-binding	Missense mutation
V172F		DNA-binding	Missense mutation
V173L		DNA-binding	Missense mutation
V173M		DNA-binding	Missense mutation
V216L		DNA-binding	Missense mutation
V216M		DNA-binding	Missense mutation
V272L		DNA-binding	Missense mutation
V272M		DNA-binding	Missense mutation
V274F		DNA-binding	Missense mutation
Y163C		DNA-binding	Missense mutation
Y220C		DNA-binding	Missense mutation
Y234C		DNA-binding	Missense mutation
Y236C		DNA-binding	Missense mutation
E171*		DNA-binding	Nonsense mutation
E198*		DNA-binding	Nonsense mutation
E204*		DNA-binding	Nonsense mutation
E287*		DNA-binding	Nonsense mutation
G266*		DNA-binding	Nonsense mutation
Q104*		DNA-binding	Nonsense mutation
Q167*		DNA-binding	Nonsense mutation
Q192*		DNA-binding	Nonsense mutation
R196*		DNA-binding	Nonsense mutation
S183*		DNA-binding	Nonsense mutation
W146*		DNA-binding	Nonsense mutation
Y205*		DNA-binding	Nonsense mutation
Y234*		DNA-binding	Nonsense mutation
Y236*		DNA-binding	Nonsense mutation
G154fs*16		DNA-binding	Frameshift mutation
P152fs*18		DNA-binding	Frameshift mutation
G334V		Tetramerization	Missense mutation
R337C		Tetramerization	Missense mutation
R337L		Tetramerization	Missense mutation
E336*		Tetramerization	Nonsense mutation
E339*		Tetramerization	Nonsense mutation
E349*		Tetramerization	Nonsense mutation
Q331*		Tetramerization	Nonsense mutation
R342*		Tetramerization	Nonsense mutation
C176F		Zinc-binding (within DNA binding domain)	Missense mutation
C176Y		Zinc-binding (within DNA binding domain)	Missense mutation
C238F		Zinc-binding (within DNA binding domain)	Missense mutation
C238Y		Zinc-binding (within DNA binding domain)	Missense mutation
H179L		Zinc-binding (within DNA binding domain)	Missense mutation
H179R		Zinc-binding (within DNA binding domain)	Missense mutation
H179Y		Zinc-binding (within DNA binding domain)	Missense mutation
E294*		Other/Outside specified domains	Nonsense mutation
E298*		Other/Outside specified domains	Nonsense mutation
E68*		Other/Outside specified domains	Nonsense mutation
Q317*		Other/Outside specified domains	Nonsense mutation
R306*		Other/Outside specified domains	Nonsense mutation
W91*		Other/Outside specified domains	Nonsense mutation
—	c.375+1G>T	—	Splice-site mutation
—	c.376-1G>A	—	Splice-site mutation
—	c.559+1G>A	—	Splice-site mutation
—	c.560-1G>T	—	Splice-site mutation
—	c.672+1G>T	—	Splice-site mutation
—	c.673-2A>G	—	Splice-site mutation
—	c.783-1G>A	—	Splice-site mutation
—	c.919+1G>T	—	Splice-site mutation
—	c.920-2A>G	—	Splice-site mutation
—	c.993+1G>T	—	Splice-site mutation
—	c.673-2A>T	—	Splice-site mutation
TP53 deletion		—	Copy number alteration

Supplementary Figure 3. Correlation between duration of treatment (DOT) and progression-free survival (PFS) in the USZ cohort. The scatter plot shows the correlation between the DOT and the PFS per patient (n = 42). The blue dotted line is the Spearman correlation test (rs =0.74; P<0.05)

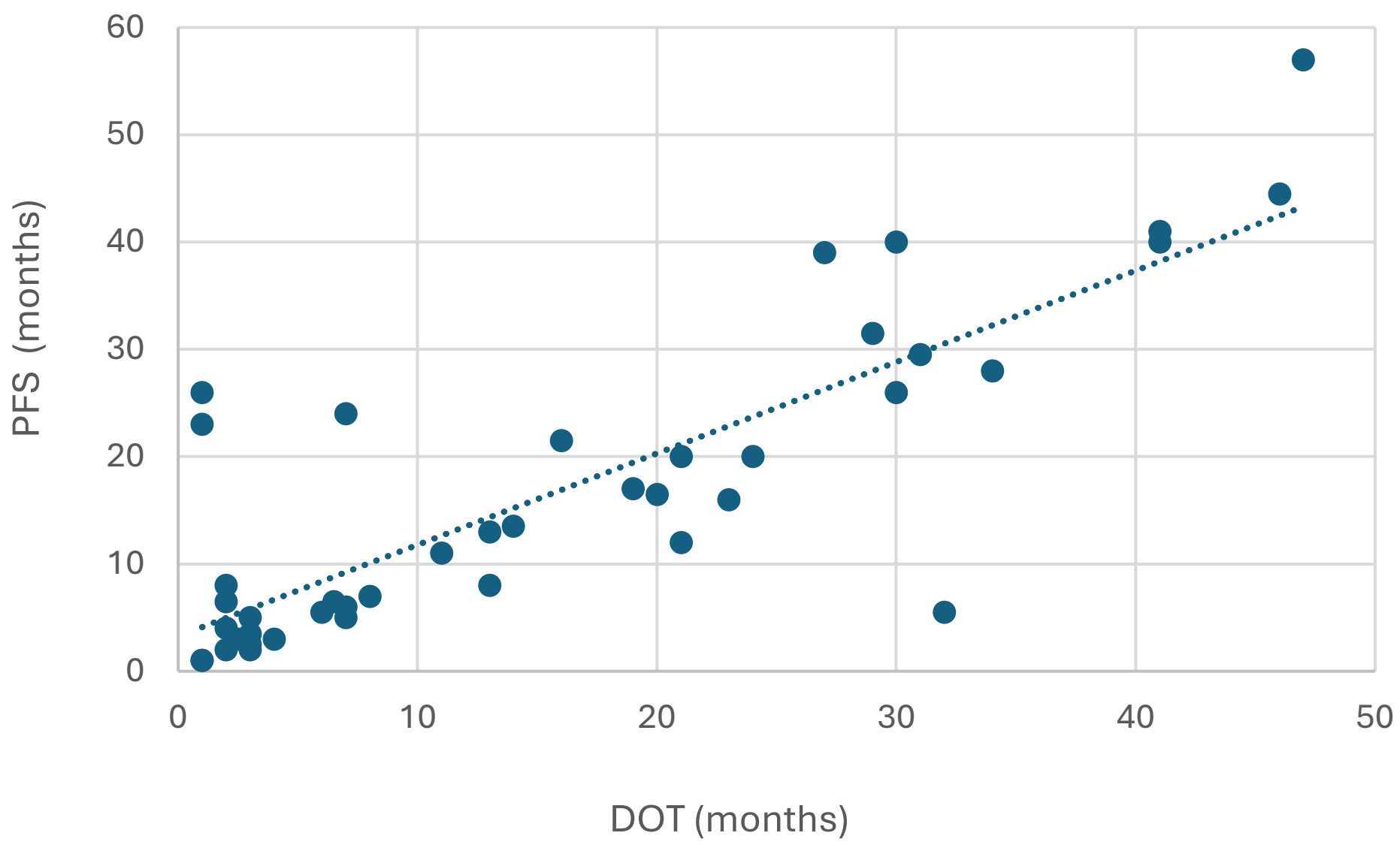

Supplementary Table 3: Duration of treatment (DOT) with first-line EGFR inhibitors (EGFRi) in the two study cohorts. Number of patients (n), median DOT, interquartile range (IQR, months), and standard deviation (SD, months), stratified by the presence or absence of specific co-alterations. Results are presented separately for osimertinib and erlotinib/afatinib in USZ (top) and FH-FMI-CGDB (bottom). Between-group comparisons were assessed using the Wilcoxon rank-sum test (WRST); p-values ≤ 0.05 were considered statistically significant.

**USZ**		**Advanced EGFR$^+$ NSCLC with co-alteration**				**Advanced EGFR$^+$ NSCLC without co-alteration**				
**Alteration**	**Treatment (1L)**	**n (%)**	**median DOT (mo)**	**IQR DOT (mo)**	**SD (mo)**	**n (%)**	**median DOT (mo)**	**IQR DOT (mo)**	**SD (mo)**	**p - value (WRST)**
MET amplification	osimertinib	0 (0)	0	-	0	22 (100)	20.5	6.5 - 30	13.0	-
	erlotinib / afatinib	1 (20)	42	-	0	4 (80)	23.5	12 - 36.5	13.2	
ERBB2 amplification	osimertinib	0 (0)	0	-	0	22 (100)	16.5	7 - 26	13.1	
	erlotinib / afatinib	0 (0)	0	-	0	5 (100)	31	16 - 42	13.7	
KRAS mutation	osimertinib	1 (5)	7	-	0	21 (95)	20	7 - 27	13.2	
	erlotinib / afatinib	0 (0)	0	-	0	5 (100)	31	16 - 42	13.7	
BRAF alteration	osimertinib	0 (0)	0	-	0	22 (100)	16.5	7 - 26	13.1	-
PIK3CA hotspot mutation	osimertinib	1 (5)	7	-	0	21 (95)	20	7 - 27	13.2	-
	erlotinib / afatinib	0 (0)	0	-	0	5 (100)	31	16 - 42	13.7	-
AKT amplification	osimertinib	0 (0)	0	-	0	22 (100)	16.5	7 - 26	13.1	-
	erlotinib / afatinib	0 (0)	0	-	0	5 (100)	31	16 - 42	13.7	-
MDM2/4 amplification	osimertinib	0 (0)	0	-	0	22 (100)	16.5	7 - 26	13.1	-
	erlotinib / afatinib	0 (0)	0	-	0	5 (100)	31	16 - 42	13.7	-
TP53 mutation	osimertinib	9 (41)	7	5 - 21	10.4	13 (59)	21	11 - 30	13.5	0.1946
	erlotinib / afatinib	0 (0)	0	-	0	5 (100)	31	12 - 42	13.7	-
CCNE1 amplification	osimertinib	0 (0)	0	-	0	22 (100)	16.5	7 - 26	13.1	-
	erlotinib / afatinib	0 (0)	0	-	0	5 (100)	31	16 - 42	13.7	-

**FH-FMI-CGDB**		**Advanced EGFR$^+$ NSCLC with co-alteration**				**Advanced EGFR$^+$ NSCLC without co-alteration**				
**Alteration**	**Treatment (1L)**	**n (%)**	**median DOT (mo)**	**IQR DOT (mo)**	**SD (mo)**	**n (%)**	**median DOT (mo)**	**IQR DOT (mo)**	**SD (mo)**	**p - value (WRST)**
MET amplification	osimertinib	17 (2)	8.2	6.7 - 17.7	11.5	687 (98)	12.4	5.7 - 22.8	14.3	0.19
	erlotinib / afatinib	15 (3)	12.1	10.7 -14.9	9.2	441 (97)	13	7.8 - 22.6	17.8	0.75
ERBB2 amplification	osimertinib	11 (2)	4	2.4 - 6.4	5.8	716 (98)	12.4	5.8 - 22.9	14.3	0.0019
	erlotinib / afatinib	7 (2)	3.8	2.6 - 9.5	8.1	454 (98)	13.1	8.1 - 22.6	17.8	0.027
KRAS mutation	osimertinib	13 (<1)	7.7	2.7 - 15.3	13.9	707 (>99)	12.3	5.7 - 22.8	14.3	0.13
	erlotinib / afatinib	8 (2)	9.5	4.1 - 18.1	15	444 (98)	13	7.8 - 22.3	17.8	0.38
BRAF alteration	osimertinib	11 (2)	11.5	7.3 - 24.5	10.8	710 (98)	12.2	5.6 - 22.3	14.3	0.86
PIK3CA hotspot mutation	osimertinib	15 (2)	6.4	3.3 - 11.7	8.4	710 (98)	12.3	5.7 - 22.7	14.2	0.043
	erlotinib / afatinib	5 (1)	3.9	3.2 -17	15.6	452 (99)	13	7.9-22.5	17.7	0.32
AKT amplification	osimertinib	15 (2)	12.1	8 - 16.2	10.4	709 (98)	12.2	5.5 - 22.6	14.3	0.91
	erlotinib / afatinib	5 (1)	9.9	1.6 - 17.1	18.5	453 (99)	13	7.8-22.4	17.7	0.46
MDM2/4 amplification	osimertinib	52 (7)	12.9	8.3 - 21	15.5	671 (93)	12.1	5.6 - 22.6	14	0.75
	erlotinib / afatinib	28 (6)	11.2	2.2 - 16.6	11.1	407 (94)	12.8	7.8 - 22.7	18	0.091
TP53 mutation	osimertinib	341 (55)	11.5	4.7 - 20	13.5	277 (45)	13.3	6.7 - 25.1	14.6	0.0066
	erlotinib / afatinib	127 (41)	10.5	4.7 - 17.1	10	185 (59)	14	9.4 - 26.9	18.2	0.0001
CCNE1 amplification	osimertinib	23 (3)	9.4	7.2 - 15.1	9.1	705 (97)	12.3	5.6 - 22.9	14.4	0.3
	erlotinib / afatinib	8 (2)	10.2	4.1 - 19.2	14.9	450 (98)	13	7.8 - 22.6	17.8	0.39

STROBE Statement—checklist of items that should be included in reports of observational studies

	Item No	Recommendation	Page No
**Title and abstract**	1	(*a*) Indicate the study's design with a commonly used term in the title or the abstract	Title page
		(*b*) Provide in the abstract an informative and balanced summary of what was done and what was found	2
**Introduction**			
Background /rationale	2	Explain the scientific background and rationale for the investigation being reported	2-3
Objectives	3	State specific objectives, including any prespecified hypotheses	3
**Methods**			
Study design	4	Present key elements of study design early in the paper	8
Setting	5	Describe the setting, locations, and relevant dates, including periods of recruitment, exposure, follow-up, and data collection	8
Participants	6	(*a*) *Cohort study*—Give the eligibility criteria, and the sources and methods of selection of participants. Describe methods of follow-up<br>*Case-control study*—Give the eligibility criteria, and the sources and methods of case ascertainment and control selection. Give the rationale for the choice of cases and controls<br>*Cross-sectional study*—Give the eligibility criteria, and the sources and methods of selection of participants	8, Fig 1
		(*b*) *Cohort study*—For matched studies, give matching criteria and number of exposed and unexposed<br>*Case-control study*—For matched studies, give matching criteria and the number of controls per case	8, Fig 1
Variables	7	Clearly define all outcomes, exposures, predictors, potential confounders, and effect modifiers. Give diagnostic criteria, if applicable	8, Fig 1
Data sources/ measurement	8*	For each variable of interest, give sources of data and details of methods of assessment (measurement). Describe comparability of assessment methods if there is more than one group	8, Fig 1
Bias	9	Describe any efforts to address potential sources of bias	8, Discussion p 6–7
Study size	10	Explain how the study size was arrived at	8
Quantitative variables	11	Explain how quantitative variables were handled in the analyses. If applicable, describe which groupings were chosen and why	8
Statistical methods	12	(*a*) Describe all statistical methods, including those used to control for confounding	8 (univariate analyses; no multivariable adjustment)
		(*b*) Describe any methods used to examine subgroups and interactions	8
		(*c*) Explain how missing data were addressed	9, Discussion p6
		(*d*) *Cohort study*—If applicable, explain how loss to follow-up was addressed<br>*Case-control study*—If applicable, explain how matching of cases and controls was addressed<br>*Cross-sectional study*—If applicable, describe analytical methods taking account of sampling strategy	
		(*e*) Describe any sensitivity analyses	Not performed

**Results**			
Participants	13*	(a) Report numbers of individuals at each stage of study—eg numbers potentially eligible, examined for eligibility, confirmed eligible, included in the study, completing follow-up, and analysed	3-4, Table 1, Spl. Table 1
		(b) Give reasons for non-participation at each stage	Fig 1
		(c) Consider use of a flow diagram	Fig 1
Descriptive data	14*	(a) Give characteristics of study participants (eg demographic, clinical, social) and information on exposures and potential confounders	3-4 Table 1, Spl. Table 1
		(b) Indicate number of participants with missing data for each variable of interest	3-4Table 1, Spl. Table 1
		(c) *Cohort study*—Summarise follow-up time (eg, average and total amount)	3-4
Outcome data	15*	*Cohort study*—Report numbers of outcome events or summary measures over time	3-4
		Case-control study—Report numbers in each exposure category, or summary measures of exposure	3-4 Spl. Fig 1
		Cross-sectional study—Report numbers of outcome events or summary measures	3-4
Main results	16	(*a*) Give unadjusted estimates and, if applicable, confounder-adjusted estimates and their precision (eg, 95% confidence interval). Make clear which confounders were adjusted for and why they were included	3-4 (unadjusted estimates; IQR and p-values reported)
		(*b*) Report category boundaries when continuous variables were categorized	3-4 Spl Fig 1
		(*c*) If relevant, consider translating estimates of relative risk into absolute risk for a meaningful time period	3-4 Spl Fig 3
Other analyses	17	Report other analyses done—eg analyses of subgroups and interactions, and sensitivity analyses	3-4 (exploratory subgroup analyses)
**Discussion**			
Key results	18	Summarise key results with reference to study objectives	5
Limitations	19	Discuss limitations of the study, taking into account sources of potential bias or imprecision. Discuss both direction and magnitude of any potential bias	5-6
Interpretation	20	Give a cautious overall interpretation of results considering objectives, limitations, multiplicity of analyses, results from similar studies, and other relevant evidence	5-6-7
Generalisability	21	Discuss the generalisability (external validity) of the study results	9
**Other information**			
Funding	22	Give the source of funding and the role of the funders for the present study and, if applicable, for the original study on which the present article is based	9

*Give information separately for cases and controls in case-control studies and, if applicable, for exposed and unexposed groups in cohort and cross-sectional studies.

Note: An Explanation and Elaboration article discusses each checklist item and gives methodological background and published examples of transparent reporting. The STROBE checklist is best used in conjunction with this article (freely available on the Web sites of PLoS Medicine at http://www.plosmedicine.org/, Annals of Internal Medicine at http://www.annals.org/, and Epidemiology at http://www.epidem.com/). Information on the STROBE Initiative is available at www.strobe-statement.org.
